# Validation of an Embedded Motion-Capture and EMG Setup for the Analysis of Musculoskeletal Disorder Risks during Manhole Cover Handling

**DOI:** 10.3390/s22020436

**Published:** 2022-01-07

**Authors:** Rémy Hubaut, Romain Guichard, Julia Greenfield, Mathias Blandeau

**Affiliations:** University Polytechnic Hauts-de-France, CNRS, UMR 8201 LAMIH, F-59313 Valenciennes, France; romain.guichard@uphf.fr (R.G.); julia.greenfield@uphf.fr (J.G.); mathias.blandeau@uphf.fr (M.B.)

**Keywords:** inertial measurement units, EMG, musculoskeletal disorder risk, ergonomics

## Abstract

Musculoskeletal disorders in the workplace are a growing problem in Europe. The measurement of these disorders in a working environment presents multiple limitations concerning equipment and measurement reliability. The aim of this study was to evaluate the use of inertial measurement units against a reference system for their use in the workplace. Ten healthy volunteers conducted three lifting methods (snatching, pushing, and pulling) for manhole cover using a custom-made tool weighting 20 and 30 kg. Participants’ back and dominant arm were equipped with IMU, EMG, and reflective markers for VICON analysis and perception of effort was estimated at each trial using a Visual Analog Scale (VAS). The Bland–Altman method was used and results showed good agreement between IMU and VICON systems for Yaw, Pitch and Roll angles (bias values < 1, −4.4 < LOA < 3.6°). EMG results were compared to VAS results and results showed that both are a valuable means to assess efforts during tasks. This study therefore validates the use of inertial measurement units (IMU) for motion capture and its combination with electromyography (EMG) and a Visual Analogic Scale (VAS) to assess effort for use in real work situations.

## 1. Introduction

Work-related Musculoskeletal Disorders (MSD) are the most common work-related problem in Europe. Almost 24% of EU workers report suffering from back pain and 22% complain of muscular pains [1]. In France, MSDs represent 87% of occupational diseases leading to a direct cost of two billion euros for the company and an indirect cost of two to seven times more [2]. The economic cost of MSDs represents between 0.5 and 2% of GDP annually for the European Union [3].

An international scientific consensus has emerged on a consensual and multifactorial approach to MSDs integrating certain physical, psychological, social and organisational characteristics of work situations [4,5,6,7,8,9]. Despite this multifactorial etiological model, preventions mainly aim to reduce biomechanical and physiological factors as these are the easiest to measure [10]. This type of approach results in prevention targeted at the sizing of the workstation, or a requirement for the workers to adapt to the characteristics of their tasks. It can lead to what Winkel and Westgaard [11] called an “*ergonomics trap*”. For instance, an improvement in working postures that does not consider the intensification of repetitive gestures fostered by a new work setup might lead to a rebound source of MSDs. The risk of developing MSDs will increase with the presence of constraints in the working environment, constraints to which the operator will not be able to provide solutions, and with the combination of the different risk factors in the same situation.

MSD assessment tools are mainly observational tools [12]. The use of wearable devices for ergonomic purposes is limited [13]. If the combination of different risk factors increases the risk of MSD, assessment tools only allow for a limited combination even of solely biomechanical factors due to measurement difficulty of some risk factors such as physical effort in the workplace [14]. Improving MSD assessment and measurement tools is a decisive move for Occupational Health and Ergonomics.

MSDs are a crucial health issue among construction workers which perform repetitive labour and intense activities exposed to weather conditions [15,16,17]. Construction workers are exposed to several MSD related risk factors such as awkward body posture, excessive vibrations, bending and twisting, working in static positions and lifting of heavy loads [17,18,19,20]. Among these workers, water supply and sanitation workers take a particular place, because their task of manhole cover opening implies lifting of heavy loads from 20 to more than 40 kg from ground level [21]. They cumulate both effort and posture, which are biomechanical risk factors of MSDs. To alleviate this biomechanical load, workers can use various tools such as a pickaxe, by pushing or pulling on the handle through leverage, or a magnet tool which allows them to directly lift the manhole cover. In this situation, comparing the MSD risk factors in the use of both tools can be a major stake for prevention purposes.

Instrument-based assessment of MSDs on the workplace can be performed by various technologies such as depth sensor cameras, inertial measurement units (IMU), and electromyography (EMG), among others [22]. Researchers have validated the effectiveness of the depth sensor camera in real-time tracking of 3D human posture [23,24]. Other studies showed the limitations of this kind of device such as self-occlusion, tracking range or occlusion by coworkers which are not suitable for an application in real work conditions [22]. An IMU sensor measures real-time acceleration and angular velocity to estimate positioning in 3D by means of sensor fusion algorithm (e.g., complementary filters, Kalman filters…). The magnetic field can also be measured but the sensor is very sensitive to magnetic disturbances (power lines, heavy metals…) and requires specific algorithms to limit this. IMU systems provide non-invasive, long-term tracking of body posture and movements [25] and have been validated by several studies for motion tracking to assess MSDs in workers [25,26,27]. IMU limitations such as the requirement of accessories (straps, belts), are sometimes uncomfortable for workers and bring a risk of sensor detachment [18]. Still, those limitations are less restrictive in the manhole handling workstation context than depth sensor occlusion. For this study, we used the IMU system created in the NOMADe project which has been validated for the quantification of classical clinical tests and for low-back pain rehabilitation evaluation [28].

Surface electromyography (EMG) is an interesting tool for workload assessment in ergonomics [29]. In human factor ergonomics, exposure concepts are derived from chemical end radiation epidemiology. Physical factors, which are part of MSD risk, are supposed to exert their effects through mechanical forces arising in the body from physical workload. The exposure-effect model focusing on mechanical exposure distinguishes external exposure and internal exposure [30]. External exposure refers to factors in the working environment, which may give rise to mechanical exposure in the body. The internal exposure comprises of forces acting on and in the body. The approach of internal exposure can be observed through electromyographic activity characterised as neuromuscular response to match the biomechanical demands. An EMG signal can be used in two different ways in ergonomics: the biomechanical approach is interested in forces and torques, whereas the physiological approach concerns muscle activation and fatigue. The relationship between EMG methodologies and MSD is mainly based on a presupposed relationship to exposure conditions or signs of fatigue estimated by EMG. Few studies demonstrate a relationship between average EMG amplitude and MSD for hand/wrist disorders [31]. Other studies show a relation between disorders and fatigue signs during work [32] or the lack of EMG-gaps during work that can predict later myalgia [30]. For the low-back region, studies tend to indicate a faster fatigue development [31,32,33,34,35].

Both IMU and EMG sensors represent a means to estimate MSD in a real-life operating environment and not in the vicinity of the laboratory. The combination of Bluetooth EMG sensors and standalone datalog systems make it suitable for out of the lab measurement. IMU are not affected by occlusion and the same datalog system can be used to avoid range tracking issues. Moreover, even if the sensors are worn on the skin, they allow the wearing of compulsory safety clothing and equipment as opposed to optical devices. Assessing MSD risk in real work conditions is a crucial prevention issue as real work conditions present a large variability of situations which cannot all be reproduced in the laboratory. Before measuring motions on site, our setup needs to be validated to a gold-standard motion-capture system. Hence, the main goal of this paper is to provide validation of an embedded motion-capture and EMG system for use in real work conditions with a population of workers.

## 2. Materials and Methods

### 2.1. Participants

Ten healthy subjects (4 females, 6 males), all members of the LAMIH UMR CNRS 8201, voluntarily participated in this study; see Table 1. Inclusion criteria were the absence of any neuro–Musculo–skeletal (low-back pain, upper limb injury, …) disorders in the last 6 months. The study was approved by the Lille University’s ethics committee (reference number: 2021-523-S97).

### 2.2. Material

#### 2.2.1. IMU Positioning

**Figure 1:**
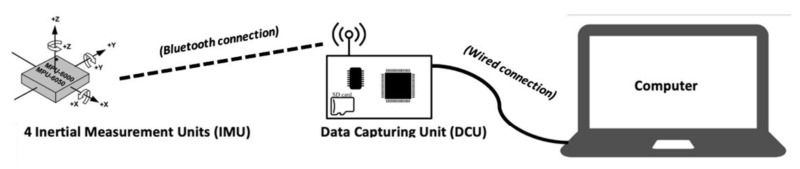
The IMU system designed by the INTERREG NOMADe consortium.

The motion-analysis system designed within the scope of consortium INTERREG: NOMADe, is composed of a Data Capturing Unit (DCU) (Dramco KUL, Ghent, Belgium) receiving the measured data of four wireless IMUs (Dramco KUL, Ghent, Belgium) (see Figure 1). The DCU acts as a controller (IMU settings and acquisition START/STOP), a preprocessor (IMU calibration and fusion of the raw data via a Madgwick filter to obtain the orientation quaternion) and storage of the acquired data. The four IMUs sensors are based on a very well-known Micro Electro Mechanical Systems (MEMS) IMU: the MPU6050, which combines a 3-axis gyroscope with a sensitivity range from 250 to 2000°/s and a 3-axis accelerometer with a sensitivity range from ±2 to ±16 g. The sample frequency can be set to 10, 20, 25, 50, or 100 Hz. The quaternions, accelerometer, and gyroscope data communicated through Bluetooth are stored in a text format file in a micro-SD card on the DCU.

**Figure 2:**
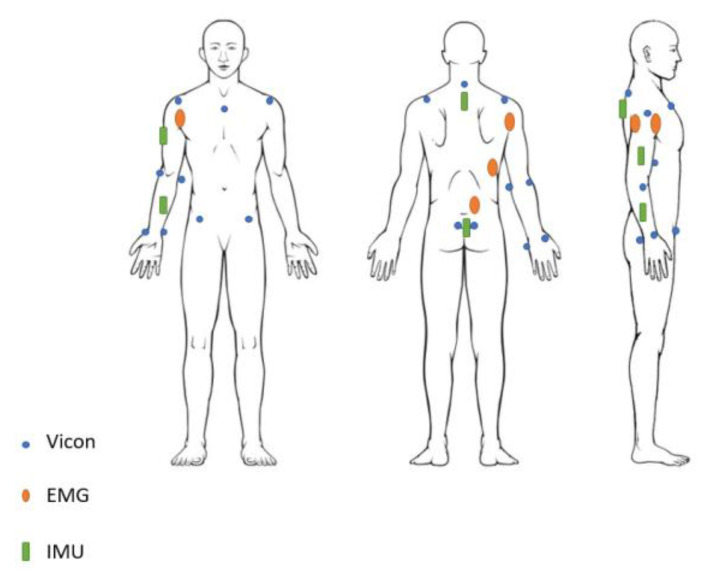
Marker placement of EMGs, IMUs, and optoelectronic markers on the subjects.

IMUs were positioned at the centre of the forearm and arm segment on the dominant arm, respectively, immediately below the C7 vertebra and between the right and left posterior superior iliac spine (see Figure 2).

#### 2.2.2. VICON Markers Positioning

The reference system for the validation of the IMU system is an optoelectronic system composed of 13 infrared cameras VICON^®^ MX (Vicon, Oxford, UK) with a sampling frequency scaled at 100 Hz. Optoelectronic markers were placed on the left and right acromia, C7, the manubrium, left and right anterior and posterior iliac spine, and the medial and lateral condyles of the dominant arm as well as the radial and ulnar styloid processes of the dominant arm (Figure 2). A reflective marker was also disposed on each IMU sensor (see Figure 2).

#### 2.2.3. EMG Positioning

EMG was measured from the subject’s dominant side (see Figure 2), deltoideus p. calvicularis (DC), deltoideus p. scapularis (DS), latissimus dorsi (LD) and erector spinae (ES) using the Wave COMETA EMG system (Cometa, Milano, Italia). The skin was shaved and cleaned, and bipolar electrodes (Ambu WhiteSensor 4535M 44 × 32 mm, Ambu, Ballerup, Denmark) were placed following SENIAM recommendations [36]. To allow the comparison of EMG signals between subjects, a normalisation based on maximal voluntary contraction was performed.

### 2.3. Protocol

**Figure 3:**
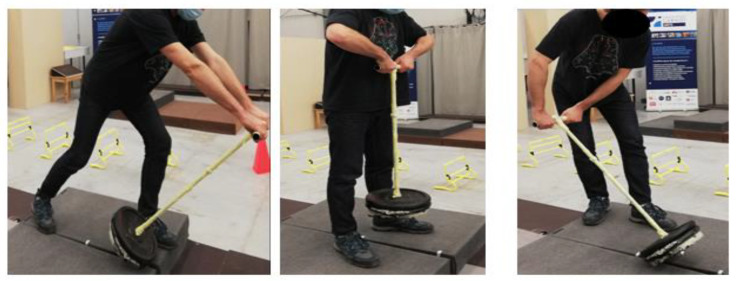
Example movements of the three motions that subjects were asked to perform in order to lift the mass: (**left**) pushing the pole, (**middle**) snatching the pole, and (**right**) pulling the pole.

The aim of the protocol explained to the participant was to lift a pole with two different masses composed of bodybuilding weights (20 and 30 kg). The participants had to use three different lifting methods: (1) pushing the pole (Ps), (2) snatching the mass with the pole (Sn), and (3) pulling the pole (Pl) (see Figure 3).

Data collection was organised such that three consecutive repetitions of the same movement were performed. Data were collected for each repetition. The lifting methods was randomly assigned at the beginning of each repetition to avoid learning effects. All three movements were firstly performed for 20 kg, followed by 30 kg; this resulted in a total of 18 movements: nine (3 repetitions × 3 lifting methods) for the 20 kg load, followed by nine for the 30 kg load. Between each repetition, a 2 min recovery phase was taken, during which participants rated the subjective necessary effort to perform each lift using a visual analogical scale (VAS). VAS was preferred to the Borg CR10 Scale [37] because of the statistical treatment allowed by the data with VAS.

Before the measurement, a warm-up and familiarisation phase of 5 min was given, within which participants were free to lift the mass in an unstructured manner.

### 2.4. Data Processing

#### 2.4.1. Synchronisation and Filtering

IMU and VICON raw data were not synchronised. Hence, a cross-correlation algorithm based on linear acceleration was developed to resynchronise all the motion data and crop all time-series to the common parts [28].

Optoelectronic data were resampled at 50 Hz to be easily compared to the IMU data. Both VICON and IMU signals were filtered by a fourth-order Butterworth low-pass filter with a cut-off frequency of 6 and 20 Hz, respectively ([38,39]).

#### 2.4.2. Linear Acceleration

Raw acceleration data measured by the IMUs include the earth’s gravity contribution. It is thus necessary to subtract the gravity vector to obtain the true linear accelerations of the IMU sensor and compare it to the VICON clusters. To do so, we expressed the acceleration vector in global coordinates using the quaternion of orientation between local and the calibration reference system computed by the DCU [40].

(1) $$Q{\left(a^{G}\right)}_{lin}=Q_{loc/G}⊗\left(Q\left(a^{loc}\right)\right)⊗\bar{Q_{loc/G}}-Q\left(g^{G}\right)$$

where $Q\left(g^{G}\right)$ is the gravitation acceleration quaternion in the global calibration frame, $Q\left(a^{loc}\right)$ is the quaternion of acceleration in the local coordinate system (linked to the IMU), $Q_{loc/G}$ is the quaternion of orientation between the local and global coordinates system, $\bar{Q_{loc/G}}$ the conjugated quaternion, and ⊗ is the operator for the quaternion product (also known as Hamilton product) [41].

Calculating back to the linear acceleration vector in the local coordinate system is obtained trivially:

(2) $$Q{\left(a^{loc}\right)}_{lin}=\bar{Q_{loc/G}}⊗\left(Q{\left(a^{G}\right)}_{lin}\right)⊗Q_{loc/G}$$

**Figure 4:**
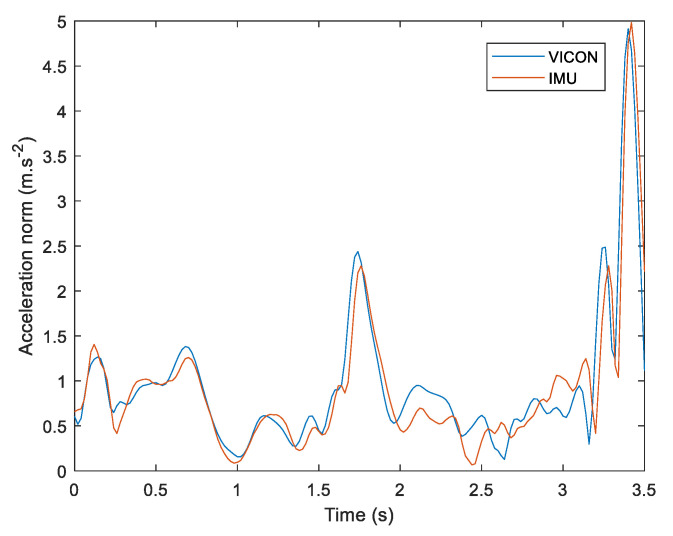
Example of linear acceleration norm during a snatching lifting method. VICON data are in blue, IMU data are in red and the maximum acceleration is at the end when dropping the weight.

After computing linear acceleration from the IMU system, the norm of this acceleration vector was computed. Linear acceleration of each IMU sensor was also computed through the VICON system by double derivation of the reflective marker’s position on each IMU sensor. An example of linear acceleration from the IMU and VICON system is presented in Figure 4 below.

#### 2.4.3. Angle Computation

Despite being very efficient as a tool for orientation computation, quaternion interpretation can be tedious. Hence, a choice was made to compare the orientation of both the IMU and VICON systems using the classical Yaw–Pitch–Roll angles (also known as *Tait-Bryan* or *Euler* angles). Yaw–Pitch–Roll angles of each IMU sensor can be deduced from $Q_{loc/G}$, the quaternion orientation. The first step is to apply the rotation matrix of the sensor from the quaternion vector [41], followed by the Yaw–Pitch–Roll angles computation using a ZYX mobile sequence [42].

The same process is applied to the VICON data from the markers applied on each segment: forearm, upper arm and pelvis. Each segment was equipped with at least 3 reflective markers whose coordinates were first used to compute a local orthogonal frame, then the segment rotation matrix. This matrix was then transformed into Yaw–Pitch–Roll angles using a ZYX mobile sequence [43].

#### 2.4.4. Time Normalisation

To be able to compare the EMG data from acquisitions with different timelines, each acquisition was normalised with respect to time. The start (resp. ending) of each acquisition was defined by a single operator for all acquisitions using the motion-capture data as the time when the subject laid their hands on (resp. removed their hands from) the pole before (resp. after) a lifting method.

Once the start and end points were defined for all acquisitions, EMG signals data were linearly interpolated using built-in MATLAB function interp1 over 1000 points.

#### 2.4.5. EMG Normalisation

EMG measurements are inherently variable between subjects due to physiological parameters (skin and fat tissue thickness above the muscle, presence of hair, sweat, etc.). Hence it is good practice to perform EMG normalisation using a maximum voluntary contraction (MVC) [44]. The MVC acquisitions were recorded prior to the lifting protocol. To compute the MVC of each muscle, the EMG signal was rectified then filtered by a 6 Hz, four order, zero-lag, low-pass Butterworth filter. Finally, a 500 ms moving average window filter was applied to the filtered signal. The MVC value was selected as the maximal value of this final signal. From this step, all EMG measurements were expressed as % MVC and associated with the appropriate muscle (see Appendix A). Data for each trial, across all muscles and participants were inspected for movement artifacts (e.g., EMG sensor moving on skin).

### 2.5. Data Analysis

The well-known Bland and Altman method [45] was used to obtain a visual representation of agreement of Yaw, Pitch, and Roll angles between the reference and the measurement system to be validated. According to [46,47], the bias and limit of agreement of the Bland and Altman method enables us to assess the correctness and accuracy of the new measurement system. An additional linear regression between the VICON and IMU systems was performed using the *corrcoef* function in MATLAB R2020b (Mathworks, Natick, MA, USA). The regression output value is the determination coefficient, *r^2^*.

Lin’s Correlation Coefficient (LCC) [48] was used in this study to obtain numerical information about agreement between measurement methods. The LCC quantifies the difference between the abscissa points of a first dataset, the ordinate of a second one, and the 45° line corresponds to perfect agreement [49]. This coefficient was chosen instead of the Intraclass Correlation Coefficient as this latter does not always give the same results depending on the computation method. LCC between 0.71 and 0.80 is *Satisfactory*, between 0.81 and 0.90 is *Fairly good*, between 0.91 and 0.95 is *Very good*, and above 0.95 is *Excellent* [46].

### 2.6. Statistic Analysis

For motion analysis, the statistical analyses were performed using the MATLAB R2020b software. The Shapiro–Wilk test was used to assess the normality of the data. At each acquisition, parameters computed with VICON and IMU data were analysed via a Student’s test to look for any statistical difference. Bonferronni corrections were used to consider the statistical repetition over all 180 acquisitions.

For EMG and VAS Analysis, the statistical analyses were performed using Statistica software (V12, StatSoft Europe, Hamburg, Germany). The Kolmogorov–Smirnoff test was used to assess the normality of the data. Repeated measures ANOVA was performed on both the data and the correlation matrix and was used to question the links between the variables. The relationship between variables for each EMG data was assessed through a Pearson’s correlation coefficient computation.

## 3. Results

### 3.1. Comparing IMU to Gold Standard

**Figure 5:**
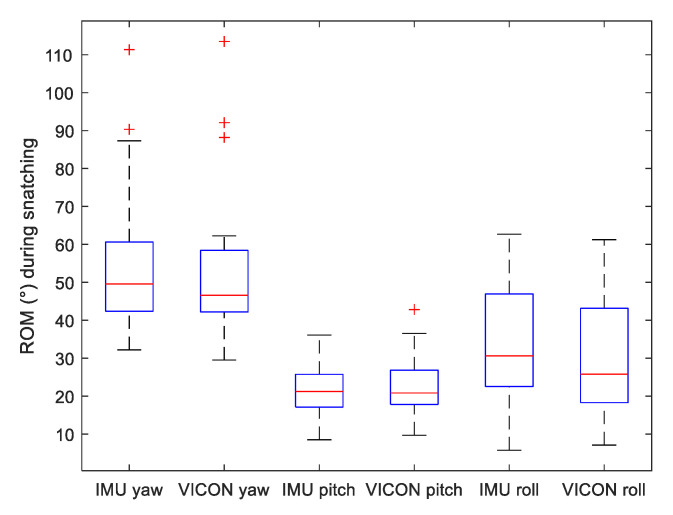
Boxplot of Range of Motion (ROM) of Yaw, Pitch and Roll angles measured by both VICON and IMU systems during *snatching* motion.

**Figure 6:**
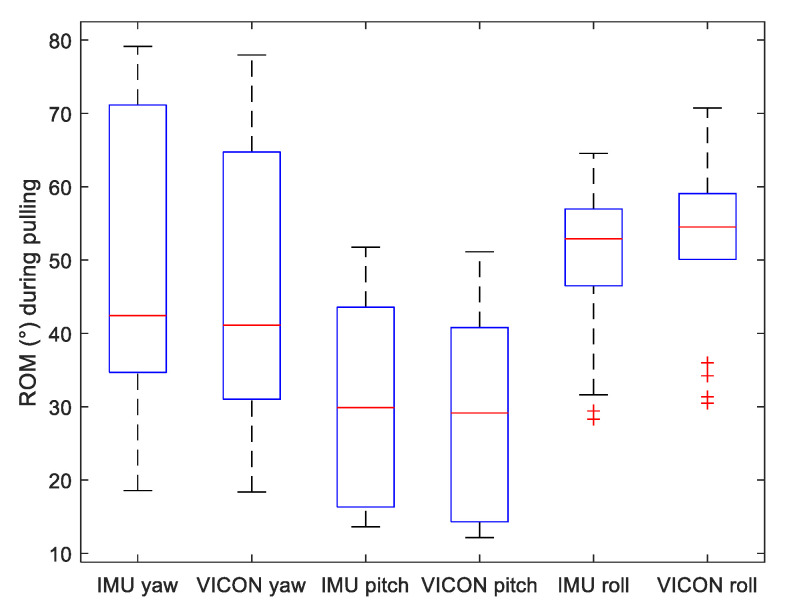
Boxplot of Range of Motion (ROM) of Yaw, Pitch and Roll angles measured by both VICON and IMU systems during *pulling* motion.

**Figure 7:**
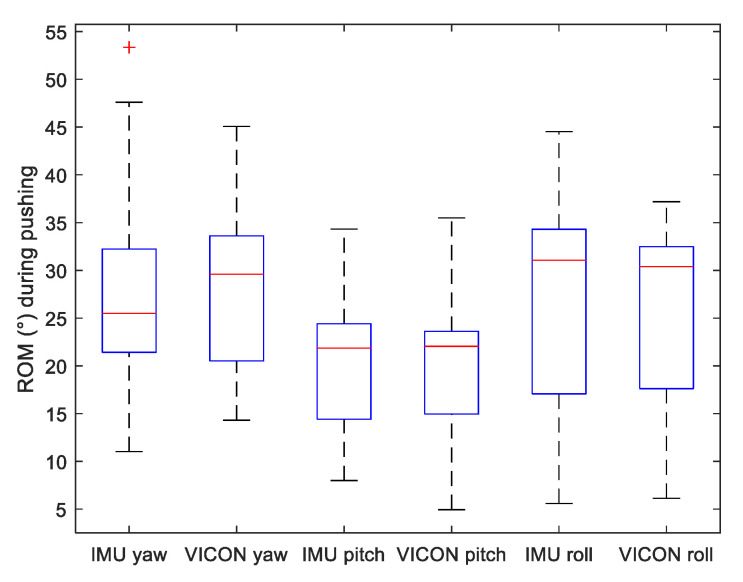
Boxplot of Range of Motion (ROM) of Yaw, Pitch and Roll angles measured by both VICON and IMU systems during *pushing* motion.

To assess motion variability of the three lifting methods, we displayed on Figure 5, Figure 6 and Figure 7 the range of motion (ROM) of the Yaw–Pitch–Roll angles computed from the IMU and the VICON system.

Results of the data analysis comparing acceleration norm and Yaw–Pitch–Roll angles between VICON and IMU systems are presented in Table 2. For all data, mean bias and LOA values are very low (especially when compared to the ROM of the above figures). Lin’s correlation coefficients are *very good* to *excellent* for all angles and *fairly good* for the acceleration norm.

No statistical difference was found between the above-mentioned parameters when analysing the effect of the VICON on the IMU system.

### 3.2. EMG Analysis

The distribution of EMG data is normal according to Kolmogorov–Smirnov test results, parametrical repeated measures ANOVA was performed on EMG data.

Table 3 shows the descriptive statistics of EMG for each muscle stratified by weight and movement.

Repeated-measures ANOVA results comparing muscle activation between each movement show statistically significant results for each muscle (DC: F_1.9_ = 30.056, *p* < 0.001; DS: F_1.9_ = 46.221, *p* < 0.001; LD: F_1.9_ = 13.395, *p* < 0.01; ES: F_1.9_ = 61.284, *p* < 0.001). Table 4 shows the effects of weight and movement on muscle activation expressed as %MVC. Only lifting methods have a significant effect on these differences.

Pairwise comparisons show non-significant differences between “pushing” and “pulling”, while there are significant differences between “pushing” and “snatching” and “pulling” and “snatching”. Post hoc analysis shows interactions between weight and movement for “snatching”, “20 kg” and “30 kg”, and the other movements (*p* < 0.001). No differences between “pushing” or “pulling”, “20 kg” and “pushing”, or “pulling” and “30 kg” were shown, except for LD which shows statistically significant differences (*p* < 0.001) between “pushing” and “pulling” “20 kg” and “30 kg”.

Table 5 presents the Pearson’s correlation coefficient between EMG signals for the LD and ES muscles, based on movement and weight. A strong positive correlation was observed between each variable for LD and ES. Correlations concerning the DC and DS muscles concern only snatching 30 and 20 kg (r = 0.7583; *p* = 0.011), pushing 30 and 20 kg (r = 0.9919; *p* < 0.0001), and pulling 30 and 20 kg (r = 0.9804; *p* < 0.0001) for DS. For DC, correlations concern pushing 20 and 30 kg (r = 0.9682, *p* < 0.0001), pushing 20 kg and snatching 20 kg (r = 0.9354, *p* < 0.0001), pushing 20 kg and pulling 30 kg (r = 0.8554, *p* = 0.02), pulling 20 kg and pushing 30 kg (r = 0.9284, *p* < 0.0001), pulling 20 and 30 kg (r = 0.9526, *p* < 0.0001), and pushing 30 kg and pulling 30 kg (r = 0.8867, *p* = 0.01).

### 3.3. VAS Analysis

VAS data showed normal distribution according to the Kolmogorov–Smirnov test; parametrical repeated measures ANOVA was performed on EMG data.

**Figure 8:**
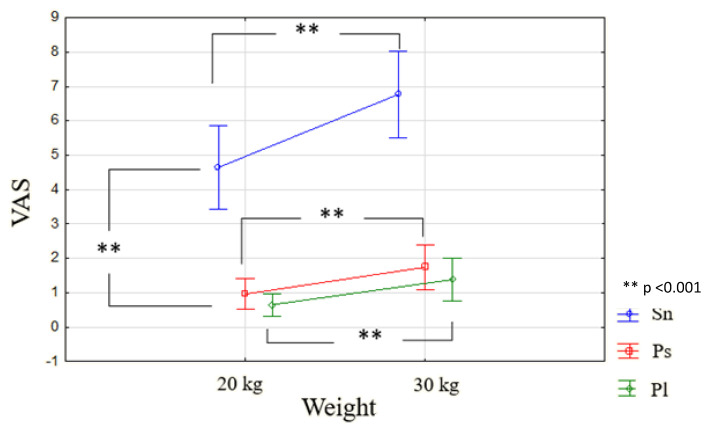
Mean of VAS depending on weight and movement.

Repeated measure ANOVA on VAS data shows a significant effect of the mass on the VAS. The means are significantly higher for 30 than 20 kg (F_1.9_ = 41.744; *p* < 0.001). We observe an effect of the movement (F_2.18_ = 95.299; *p* > 0.05): pairwise comparisons show non-significant difference between pushing and pulling while there are significant differences between pushing and snatching (1.35 vs. 5.70; *p* < 0.001) and pulling and snatching (1.02 vs. 5.70; *p* < 0.001). We observed an interaction between the weight and movement (F_2.18_ = 6.0825; *p* < 0.01). We noted on Figure 8 as well as on the simple effects that for the “snatching” gesture, the “30 kg” condition is higher than the “20 kg” (*p* < 0.00001). This is also the case for the “pushing” condition, but was less clearly observed. On the other hand, for the “pulling” condition, there is no difference between the “20 kg” and “30 kg” conditions. There was a positive correlation between VAS and EMG for Snatching 20 kg (r = 0.7889, *p* = 0.007) for DC.

## 4. Discussion

The main goal of this paper was the validation of an embedded motion-capture system for use in a real work situation. Motion capture was performed by means of IMU and EMG sensors, which were compared to optoelectronic motion-capture data and VAS rating the effort of each trial, respectively.

### 4.1. Limitations

Some limitations are present in our study. First of all, the fact of using two different motion-capture systems meant that synchronization between the IMU and VICON data at 50 Hz was necessary. Despite previous validation of this method and visual inspection of all acquisitions, small delays between the data can still persist. At 50 Hz, a two-frame delay represents 40 ms which is enough to yield the small values of Lin’s concordance coefficient on rapidly changing data such as acceleration (Table 2). Future works will be dedicated to correcting this limitation. The current solution considered is the design of a synchronisation bench which will be linked as an analog device to the VICON system and will move the IMU following a known trajectory.

The motion variability was very important, and this was due to the three lifting methods and the participants’ anthropometrical variability. Indeed, participants were free to adapt the lifting method to their anthropological parameters. For instance, during snatching, a tall subject would lift the mass with his/her hands above the horizontal bar of the mass when a smaller subject would rather lift it with his/her hands below the bar. Although our results are positive on the use of this embedded motion-capture setup, further research with larger sample size including differences in anthropometry would improve the validation statement.

One potential source of error in the orientation estimation of the segments could be due to the well-known soft tissue artefacts (STA) and wobbling masses. STA represent an important error source both for IMU and VICON systems in differing ways due to variations in sensor position. In optoelectronic systems, reflective markers are commonly positioned on bone points to reduce skin displacement during motions whereas IMU sensors are usually placed in the middle of the segments to avoid measuring the displacement of two segments at the same time. Naturally, this type of error will depend on the anthropometry of the subjects. Nevertheless, a preliminary study [50] showed little effects of STA and wobbling masses when accelerations were low (which is the case for the different lifting methods).

Lastly, better precision could have been obtained by using an IMU with a three-axis magnetometer sensor (e.g., MPU9250). However, the use of a magnetometer exposes measurements to an increased sensitivity to magnetic disturbance [51], which can be very important in the current context of our study (high-power cables, heavy metals, construction machines, etc.).

### 4.2. Motion-Capture Validation

The main goal of this section was to test the reliability, reproducibility, and robustness of the IMU sensors on various segments and motions. It was not to quantify the motion variability between the three (obviously different) lifting methods but to provide evidence that the embedded motion-capture set was precise enough to be used in the inherent variability of the real work situations.

It is important to state that we focused on segment orientation rather than joint angles and this for two main reasons. First of all, biomechanical modelling of the upper limbs kinematics (especially at the elbow level) is still not standardised for the use of IMU as it is for optoelectric systems [52]. This disparity creates a lot of errors in the validation of IMU systems against state-of-the-art motion analysis technics [53,54]. The second reason is linked to the field of application of our IMU system in the prevention of MSD in real work situations (i.e., away from the motion-capture lab). In ergonomics, one gold-standard method to perform posture analysis and MSD assessment is to use survey methods such as RULA (Rapid Upper Limb Assessement) which is amongst the most renowned [55]. This method is not focused on range of motion of the joint but of the individual segments, which is why our validation method is coherent with the field of applied ergonomics.

Despite the high variability in the results due to the three different lifting motions, the IMU system precision is coherent with previous scientific works. In [53], the authors compared the angle ROM obtained from a low-cost IMU system (Perception Neuron, Noitom, Miami, FL, USA) with a classical optoelectronic system and the proprietary software. The Bland–Altman method resulted in bias values between −11.1° and 8.1°, $\mathrm{LOA}\in \left[\begin{array}{cc} -19{}^{\circ} & 26{}^{\circ} \end{array}\right]$ for the IMU placed on the upper limbs, which clearly shows the superiority of our system. In [54], the same authors compared gold-standard motion capture with a more renowned IMU system (MVN, Xsens technologies, Enschede, The Netherlands). Once again, the Bland–Altman method resulted in bias values of the upper limbs IMU between −1° and 0.6°, $\mathrm{LOA}\in \left[\begin{array}{cc} -5{}^{\circ} & 4{}^{\circ} \end{array}\right]$.

Hence, our motion-capture results presented in Table 2 are coherent with the existing state of the art in embedded motion capture. Future work could be dedicated to injecting the measured kinematics into human body modelling [56] to allow the estimation of joint torques during the different motions.

### 4.3. EMG and VAS a Complementary Set of Measurements

EMG data analysis shows significant differences of muscular activity based on the movement performed by the subject and the weight lifted. Statistical analysis shows an interactional effect of movement and weight over the activity of different muscles. Correlation analysis shows us the muscles whose variations are strongly linked with the task rather than the motor strategy of the subject. This is particularly relevant for back muscles as they are less assumed by motor strategy variations than the shoulder muscles. This is particularly important for efforts assessment in real work situations where effort assessment on the shoulders can be linked to the strategy of the workers depending on the specificities of the situation, when back muscles activity will be linked with the task itself. As already shown in various studies, EMG is a relevant choice to compare muscular activity during the use of various tools and different tasks [57,58]. Considering EMG data as a continuous value allows for a more complex analysis than a simple Exposure Variation Analysis (EVA) treatment [29] which limits the use of statistics to non-parametrical tests. In our protocol, EMG data can be compared between various tasks or working conditions. As far as we know, no other study offers such a statistical treatment of EMG in the workplace. One of the main causes could be the difficulties of assembling a sufficient sample of workers.

An interesting point is the link between the effort measured by the EMG and the perceived effort assessment by the subject using VAS. VAS measures of perceived effort by the subject also show statistically significant differences based on the movement and on the weight performed by the subject. VAS appears to be more sensitive to weight than the muscular activity. This can be explained by the perception of the weight lifted by the subjects. In correlational analysis, we note a strong relation between VAS and DC in snatching tasks. This may be an order effect of our protocol. It also can be linked with the fact than this muscle is not usually involved in manual handling tasks. In real work situations, perception of the weight of the objects lifted by a worker is rarely so clear. VAS shows the same differences as the EMG. In a measurement method during a real work situation, VAS can be used as a back-up measurement in case of loss of EMG signal due the workers’ movements.

When using a new measurement method, it is necessary to validate it and compare these results with a gold standard and estimate the agreement between these two measurement methods. The particularity of work situations where professional tools and gestures are often unique to a company practice can make it difficult to find this reference basis. This study allows us to have a reference basis to compare EMG values with the future EMG found in real work situations. The use of EMG measurement will allow comparisons such as the tools used for the opening of a manhole cover, and the muscle activity around various factors such as the experience of the worker performing the movement and to compare the effects of these strategies on muscle activity. EMG and motion analysis can also be used for tool sizing, and helping to adjust the height of the handle regarding the efforts needed to achieve the task [59]. This method also provides a means of treating manual handling which is significantly above the ergonomic recommendations where the configuration and variability of the situation does not always allow the deployment of tools other than manual tools.

## 5. Conclusions

The embedded motion-capture and EMG setup developed in this study has been positively validated for the real work condition study of manhole-cover handling. Our work shows that our embedded motion-capture set is precise enough to be used in real work situations. Our EMG data analysis shows than EMG is a relevant choice to compare muscular activity during the use of various tools and different tasks. EMG analysis as a continuous variable allows the use of parametrical statistical tests. Moreover, there is an interesting link between EMG measurement and the self-perception of effort rated by a VAS. Future work will be dedicated to using this setup to assess the impact of work conditions (i.e., cover weight, tools for lifting methods, etc.) on both the motion and effort perception of lifting.

## Figures and Tables

**Table 1:** Characteristics of the participants (mean + standard deviation).

Age (Years)	Body Mass (kg)	Height (m)
31.7 ± 10.5	73.6 ± 15.9	1.74 ± 0.08

**Table 2:** Results computed from the Bland–Altman method and Lin’s coefficient of sensor validation. All values are averaged over acquisitions with standard deviation in parentheses.

Parameter Name	Acceleration Norm (m/s^2^)	Yaw Angle (°)	Pitch Angle (°)	Roll Angle (°)
bias	−0.006 (0.176)	0.224 (2.1)	0.139 (1.599)	−0.864 (0.237)
lower LOA	−0.682 (0.465)	−3.180 (2.812)	−2.397 (1.458)	−4.474 (3.587)
upper LOA	0.640 (0.407)	3.628 (2.524)	2.676 (2.200)	2.745 (2.384)
precision	0.281 (0.206)	2.127 (1.240)	1.578 (0.824)	2.226 (1.691)
r^2^	0.768 (0.173)	0.976 (0.028)	0.924 (0.092)	0.951 (0.084)
Lin’s CC	0.859 (0.136)	0.958 (0.067)	0.908 (0.107)	0.928 (0.121)

**Table 3:** Mean and standard deviation for each movement for deltoideus p. calvicularis (DC), deltoideus p. scapularis (DS), latissimus dorsi (LD), and erector spinae (ES).

		DC		DS		LD		ES	
Weight	Movement	M	Sd	M	Sd	M	Sd	M	Sd
20 kg	Sn	4.648	0.540688	22.5236	4.213061	12.0361	3.586532	22.0377	2.895057
	Ps	0.966	0.198473	10.422	3.084158	6.4182	2.028903	15.1535	2.592818
	Pl	0.656	0.144846	5.47851	1.961944	10.6167	2.199019	15.3425	2.173868
30 kg	Sn	6.77	0.560089	30.5888	5.130576	13.696	3.930682	25.1647	2.787131
	Ps	1.744	0.291895	9.3448	2.903456	5.82487	1.767021	14.0036	2.3651873
	Pl	1.384	0.275521	4.4531	1.159783	9.7306	2.585124	14.837	1.968479

Note: M: Mean; Sd: Standard Deviation; Sn: Snatching method; Ps: Pushing method; Pl: Pulling method.

**Table 4:** ANOVA on EMG (%MVC).

Muscle	Source of Variation	df	F
DC	Weight	1	2.01676
	Movement	2	26.57875 **
	Weight × Movement	2	3.97352 *
DS	Weight	1	3.44022
	Movement	2	19.4128 **
	Weight x Movement	2	8.23358 **
LD	Weight	1	0.04502
	Movement	2	6.23308 **
	Weight × Movement	2	8.42081 **
ES	Weight	1	1.08632
	Movement	2	21.99597 **
	Weight × Movement	2	4.4893 *

Note: * *p* < 0.05; ** *p* < 0.01.

**Table 5:** Correlation table for LD and ES.

Muscle	Variable	1	2	3	4	5	6
LD	1. Sn 20kg						
	2. Ps 20kg	0.9162 **					
	3. Pl 20kg	0.8219 **	0.8799 **				
	4. Sn 30kg	0.9903 **	0.9562 **	0.8294 **			
	5. Ps 30kg	0.8842 **	0.9954 **	0.8802 **	0.9318 **		
	6. Pl 30kg	0.785 **	0.8831 **	0.9888 **	0.8042 **	0.8911 *	
ES	1. Sn 20kg						
	2. Ps 20kg	0.7427 *					
	3. Pl 20kg	0.6449 *	0.8692 **				
	4. Sn 30kg	0.9201 **	0.8197 **	0.8452 **			
	5. Ps 30kg	0.6911 *	0.9225 **	0.9225 **	0.8618 **		
	6. Pl 30kg	0.7156 *	0.8237 **	0.9197 **	0.8325 **	0.7822 **	

Note: * *p* < 0.05; ** *p* < 0.01.

**Figure A1:**
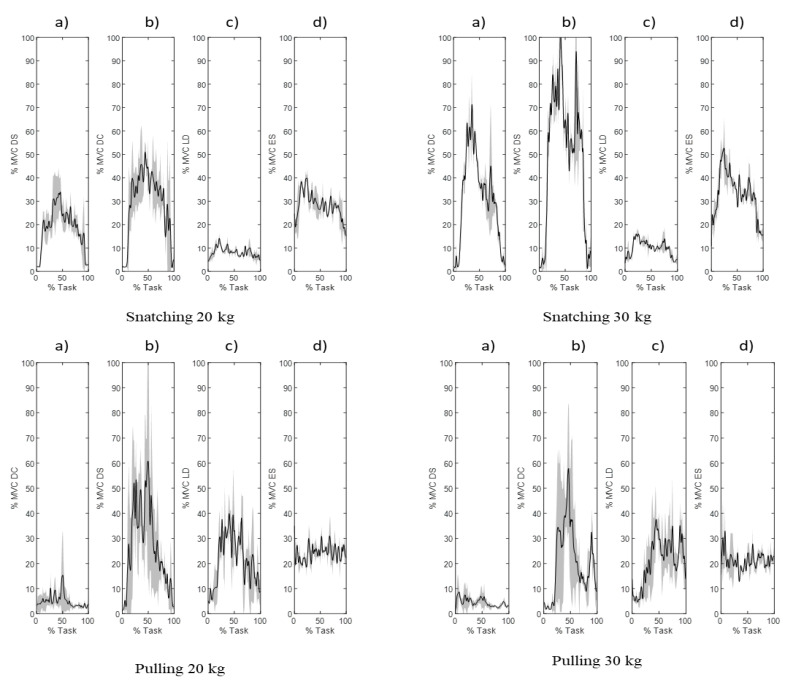
EMG signals for subject 1 for the (**a**) DC muscle; (**b**) DS muscle; (**c**) LD muscle, and (**d**) ES muscle.

## Data Availability

The data presented in this study are available on request from the corresponding author.

## Appendix A. EMG Signal Illustration

Appendix A illustrates the normalised EMG measurements for the three repetitions of snatching at 20 and 30 kg and pulling at 20 and 30 kg on one subject.
